# Remote‐Controllable Interfacial Electron Tunneling at Heterogeneous Molecular Junctions via Tip‐Induced Optoelectrical Engineering

**DOI:** 10.1002/advs.202305512

**Published:** 2023-12-06

**Authors:** Jinhyoung Lee, Eungchul Kim, Jinill Cho, Hyunho Seok, Gunhoo Woo, Dayoung Yu, Gooeun Jung, Hyeon Hwangbo, Jinyoung Na, Inseob Im, Taesung Kim

**Affiliations:** ^1^ School of Mechanical Engineering Sungkyunkwan University (SKKU) Suwon‐si Gyeonggi‐do 16419 Republic of Korea; ^2^ AVP process development team Samsung Electronics Cheonan‐si Chungcheongnam‐do 31086 South Korea; ^3^ SKKU Advanced Institute of Nanotechnology (SAINT) Sungkyunkwan University Suwon 16419 Republic of Korea; ^4^ Department of Nano Science and Technology Sungkyunkwan University Suwon 16419 Republic of Korea; ^5^ Park Systems Corp R&D Center Suwon 16229 Republic of Korea; ^6^ Department of Nano Engineering Sungkyunkwan University Suwon 16419 Republic of Korea

**Keywords:** photo‐induced force microscopy, Kelvin probe force microscopy, molecular tunneling junction, interfacial charge transfer, DFT calculation

## Abstract

Molecular electronics enables functional electronic behavior via single molecules or molecular self‐assembled monolayers, providing versatile opportunities for hybrid molecular‐scale electronic devices. Although various molecular junction structures are constructed to investigate charge transfer dynamics, significant challenges remain in terms of interfacial charging effects and far‐field background signals, which dominantly block the optoelectrical observation of interfacial charge transfer dynamics. Here, tip‐induced optoelectrical engineering is presented that synergistically correlates photo‐induced force microscopy and Kelvin probe force microscopy to remotely control and probe the interfacial charge transfer dynamics with sub‐10 nm spatial resolution. Based on this approach, the optoelectrical origin of metal–molecule interfaces is clearly revealed by the nanoscale heterogeneity of the tip‐sample interaction and optoelectrical reactivity, which theoretically aligned with density functional theory calculations. For a practical device‐scale demonstration of tip‐induced optoelectrical engineering, interfacial tunneling is remotely controlled at a 4‐inch wafer‐scale metal–insulator–metal capacitor, facilitating a 5.211‐fold current amplification with the tip‐induced electrical field. In conclusion, tip‐induced optoelectrical engineering provides a novel strategy to comprehensively understand interfacial charge transfer dynamics and a non‐destructive tunneling control platform that enables real‐time and real‐space investigation of ultrathin hybrid molecular systems.

## Graphical abstract

Tip‐induced optoelectrical engineering at molecular junction is presented, which has not been previously achieved due to the critical limitations of existing spectroscopy methods. The heterogeneous interfacial charge transfer dynamics are completely revealed via synergistic correlation of PiFM and KPFM. It provides the interfacial tunneling control platform that enables the non‐destructive real‐time and ‐space investigation of ultrathin hybrid molecular junction systems.

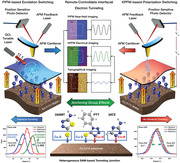

## 1 Introduction

Molecular electronics enables functional electronic behavior using single molecules or molecular self‐assembled monolayers (SAMs). This has attracted significant attention owing to the emergence of flexible electronics,^[^ ^1^ ^]^ solar cell,^[^ ^2^ ^]^ LED,^[^ ^3^ ^]^ and battery,^[^ ^4^ ^]^ and physical limitations of semiconductor device miniaturization.^[^ ^5^ ^]^ Over the past two decades, molecular junction systems have evolved into platforms that enable atomic‐scale electrical investigations of molecules, including the energy gap,^[^ ^6^ ^]^ I‐V characteristics,^[^ ^7^ ^]^ energy conversion,^[^ ^8^ ^]^ and molecular conductance.^[^ ^9^ ^]^ As the electrical characterization of molecules has become more important for accomplishing the target electronic functionalities, various electrochemical spectroscopic methods and molecular junction structures have emerged to investigate the charge transfer dynamics in molecular electronics.^[^ ^10^ ^]^

In previous studies, various molecular junction structures were constructed using scanning tunneling microscopy break junctions,^[^ ^11^ ^]^ conductive atomic force microscopy (CAFM),^[^ ^12^ ^]^ mechanically controllable break junctions,^[^ ^13^ ^]^ carbon nanotube junctions,^[^ ^14^ ^]^ molecular wire junctions,^[^ ^15^ ^]^ moldable liquid,^[^ ^16^ ^]^ and tip‐enhanced Raman spectroscopy (TERS).^[^ ^17^ ^]^ However, these conventional molecular junction structures have several limitations including mechanical instability,^[^ ^18^ ^]^ low junction yield,^[^ ^19^ ^]^ and interface charging / trapping effects.^[^ ^20^ ^]^ Recently, the low yield problem has been solved with nanoparticle contacts^[^ ^21^ ^]^ and improvements in mechanical stability have been reported through interstitial mixing.^[^ ^22^ ^]^ However, since the interface charging/trapping still exists in junction contact interfaces,^[^ ^23^ ^]^ the utilization of molecular junctions is constrained because the absence of well‐defined design rules for molecular structures cannot consistently present stable electrical properties. Notably, even minor morphological alterations can lead to significant fluctuations in the charge transfer dynamics within the conventional molecular junction structures.^[^ ^24^ ^]^ The 3D spatial correlation of the morphological and electrochemical reactivities of molecular junction is impossible using conventional spectroscopic techniques and molecular junction structures.^[^ ^25^ ^]^ Therefore, the development of noncontact molecular junction structures and high‐resolution electrochemical spectroscopic methods has emerged as a key challenge for overcoming these limitations.

Recently, TERS has emerged as a promising spectroscopic technique for non‐destructive characterization of the morphological and electrochemical properties of molecular electronics, which combines tip‐scanning imaging with Raman spectroscopy.^[^ ^20^, ^26^ ^]^ By combining single‐molecule sensitivity with ultrahigh spatial resolution, TERS provides a comprehensive and in‐depth understanding of organic electronics.^[^ ^17^, ^27^ ^]^ In addition, several studies have constructed a nanoscale tunneling gap between the TERS tip and molecule to avoid direct contact between the metal electrode and molecule.^[^ ^28^ ^]^ TERS significantly improves the sensitivity of Raman spectroscopy by employing a sharp metallic tip to locally amplify the electromagnetic field of the incident laser,^[^ ^29^ ^]^ which enhances the localized Raman scattering at the tip–sample junction. However, TERS generates interference from far‐field scattered photons, which enhances the background signal in the localized tip apex region.^[^ ^30^ ^]^ Because the overwhelming near‐field TERS signal obscures the contrast between near‐ and far‐field signals, the optically driven background signal hinders the widespread use of TERS and reliable near‐field probing.^[^ ^31^ ^]^

To overcome these limitations, a synergistic correlation of photo‐induced force microscopy (PiFM) and Kelvin probe force microscopy (KPFM) is proposed to remotely control the interfacial tunneling dynamics, enabling real‐time and real‐space investigations of ultrathin hybrid molecular electronic systems with a sub‐10 nm spatial resolution.^[^ ^32^ ^]^ Based on remote‐controllable interfacial charge transfer under a tip‐induced optoelectrical field, the interfacial charging effect and far‐field scattering interference, which are critical limitations of metal–molecule–metal junctions, are simultaneously overcame. The nanoscale heterogeneity of the interfacial tunneling dynamics was observed via 3D spatial mapping of the PiFM signal, work function, and tip–sample interaction. The underlying mechanism of the observed heterogeneous interfacial tunneling dynamics was theoretically validated based on density functional theory (DFT) calculations and theoretical tip–sample interaction modeling. Consequently, the results demonstrate that interfacial tunneling dynamics can be remotely controlled at SAMs‐based tunneling junctions via tip‐induced optoelectrical engineering, providing new opportunities for next‐generation molecular‐based optoelectronic devices.

## 2 Results and Discussion

### 2.1 Remote‐Controllable Interfacial Tunneling via Tip‐Induced Optoelectrical Engineering

**Figure 1:**
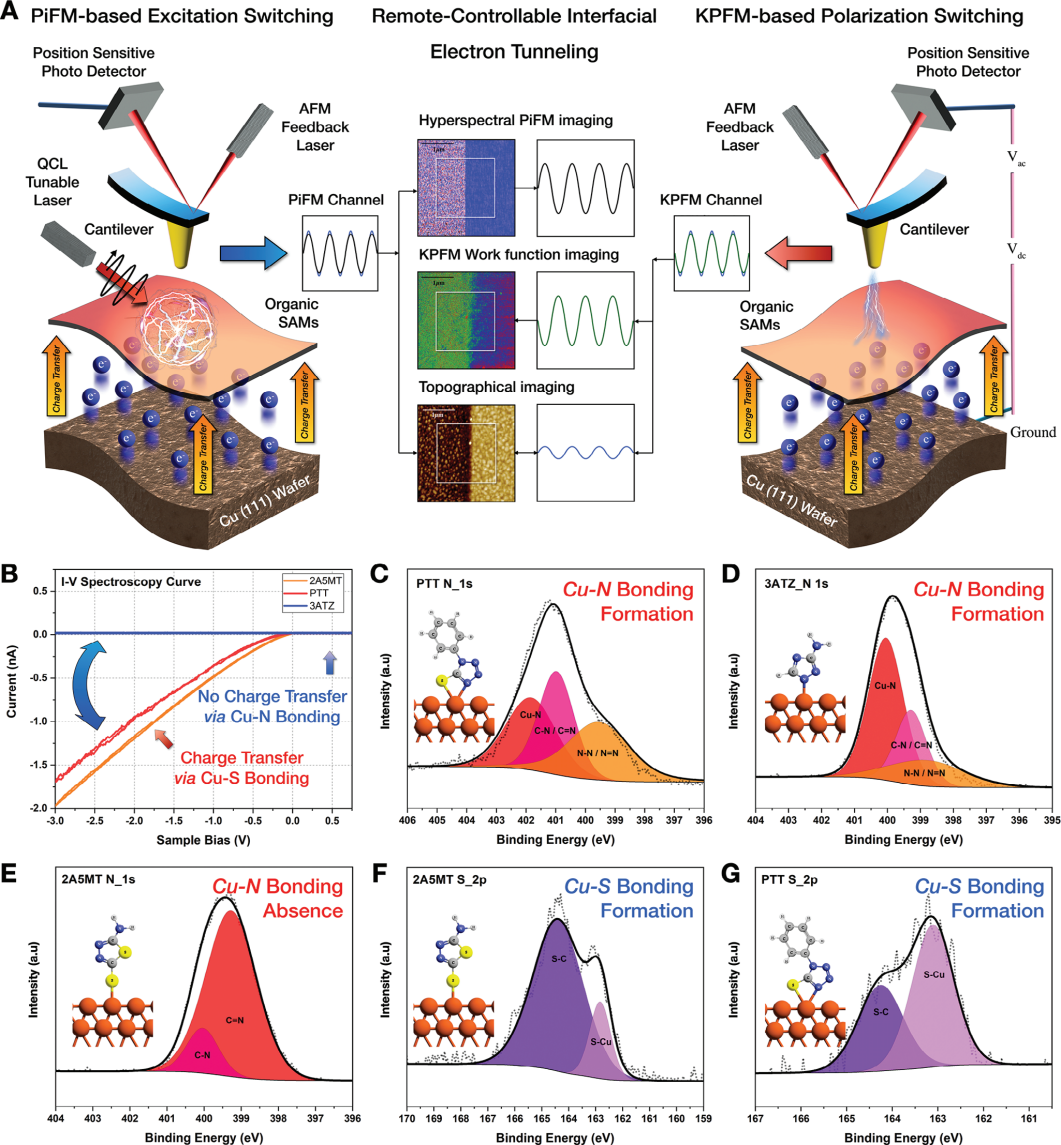
Remote‐controllable interfacial tunneling dynamics at SAMs‐based tunneling junctions via tip‐induced optoelectrical engineering. A) Schematic diagram of experimental design of tip‐induced optoelectrical engineering based on PiFM IR‐excitation and KPFM polarization. B) CAFM current‐voltage curve of sulfur‐anchored (2A5MT, PTT) and nitrogen‐anchored (3ATZ) self‐assembled domain, exhibiting the intrinsic charge transfer properties of molecule. XPS N 1s spectra of C) PTT, D) 3ATZ, E) 2A5MT, and S 2p spectra of F) PTT, G) 2A5MT, which clarifies the evident formation of covalent bonding, activating the hybrid tunneling states at the metal‐molecule interfaces.

A remote interfacial tunneling control platform was constructed at SAMs‐based tunneling junctions using PiFM and KPFM to remotely control the interfacial tunneling dynamics in metal–molecule heterostructures. This experimental design overcomes the conventional limitations of molecular junction structures, as shown in **Figure** **1**A. Based on the tip‐induced optoelectrical control platform setup, direct contact between the Platinum atomic force microscope (AFM) tip and the SAMs surface was avoided by constructing a molecular junction structure with a tunneling gap (air, 5 nm) that acted as a dielectric barrier between the tip apex and the molecule (Figures S1 and S2, Supporting Information). Because PiFM measures sample polarizability based on the photo‐induced force (PiF) gradient at the tip–sample junction, in contrast to other tip‐enhanced near‐field optical microscopy techniques, PiF‐based near‐field detection enables reliable near‐field probing without far‐field background contribution. In addition, KPFM and PiFM experiments were conducted non‐destructively in non‐contact states, which were experimentally validated in the absence of deformation and contamination at the AFM tip (Figure S3, Supporting Information). In contrast, the CAFM I‐V spectroscopy in Figure 1B, measured to investigate the intrinsic electrical characteristics of SAMs through a conventional CAFM junction, was measured in the contact state.

To experimentally demonstrate the remote‐switchable interfacial charge transfer dynamics, a metal–molecule heterostructure was constructed through SAMs formation on a Cu (111) surface, for which three heterocyclic compounds were selected: 2‐amino‐5‐mercapto‐1,3,4‐thiadiazole (2A5MT), 3‐amino‐1,2,4‐triazole (3ATZ), and 1‐phenyl‐1*H*‐tetrazole‐5‐thiol sodium salt (PTT). The anchoring groups of the selected heterocyclic compounds were adsorbed on the Cu (111) surface via different heteroatoms: sulfur atoms for 2A5MT, nitrogen atoms for 3ATZ, and nitrogen and sulfur atoms for PTT. Despite the similarities between the nitrogen and sulfur atoms in the anchoring group, these heteroatoms showed a distinguishable atomic charge distribution within each anchoring group, leading to heterogeneity in the interfacial charge transfer dynamics. To further strengthen the demonstration of remote tunneling control, CAFM I–V curve measurements were performed at a metal (Cu)–molecule–AFM tip (Pt) contact junction to obtain the intrinsic charge transfer properties, as shown in Figure 1B. Heterogeneous tunneling dynamics at the nanoscale were experimentally observed with CAFM I‐V characteristics, which were obtained as −0.502 nA for 2A5MT and −0.382 nA for PTT at a sample bias of −1 V, whereas 3ATZ exhibited a negligible current (0.003 nA). Such distinguishable tunneling dynamics are attributed to intramolecular electron‐pulling and ‐pushing effects, which are predominantly governed by the atomic charge distribution of the anchored heteroatom. In the sulfur‐anchored domain, electron‐pulling effects efficiently stimulated interfacial charge transfer through Cu─S bonding, whereas charge transfer in the nitrogen‐anchored domain was mainly blocked by electron‐pushing effects, which generated an interfacial electron‐repelling barrier.

To experimentally observe the formation of hybrid tunneling states at the metal–molecule interfaces, X‐ray photoelectron spectroscopy (XPS) was conducted. In the N 1s spectrum of PTT, the Cu─N peak (408.8667 eV) indicates adsorption on the copper surface. In addition, N─N or N═N (399.1411 eV) and C─N or C═N peaks (401.1762 eV) are presented in Figure 1C. Figure 1D shows the N 1s spectrum of 3ATZ; Cu─N (400.0297 eV), N─N or N═N (399.2490 eV), and C─N or C═N (399.3037 eV) peaks are observed. In contrast, because the N─N bond intensity of the 2A5MT N 1s peak (Figure 1E) was relatively weak or nearly absent, it was experimentally confirmed that 2A5MT was adsorbed on a monolayer scale. The N 1s spectra of 3ATZ and PTT show similar XPS peak configurations originating from homogeneous Cu─N bonds and similar heterocyclic ring configurations. As shown in Figure 1F, in the S 2p spectrum of 2A5MT, a Cu─S peak (162.8322 eV) corresponding to copper adsorption and a C─S peak (164.3906 eV) were observed. Similarly, in the S 2p spectrum of PTT (Figure 1G), Cu─S (163.1097 eV), and C‐S peaks (164.2515 eV) peaks were observed. This is because of the homogeneous Cu─S bonds and similar heterocyclic ring configurations. The XPS results revealed that the heterocyclic compound was strongly adsorbed on the Cu (111) surface. Cu─S and Cu─N bonding occurred in 2A5MT and 3ATZ, respectively, and Cu─S and Cu─N bonding occurred in PTT. Fourier‐transform infrared spectroscopy characterization of the intramolecular covalent bonding and optical heterogeneity between the SAMs region and the bare Cu (111) wafer additionally supported the reliable formation of interfacial covalent bonds (Figure S4, Supporting Information).

Based on XPS O 1s peak results (Figure S5, Supporting Information), all organic SAMs indicates a distinctive decrease in O 1s peak intensity, which confirms that the organic SAMs formation effectively prevents oxidation and excellent SAMs packing quality. As SAM quality largely depends on the work function of bottom electrode and can affect charge transfer property of junction,^[^ ^33^ ^]^ work function of bottom electrode was measured. Work function of Cu (111) electrode (Figure S6, Supporting Information) was measured as 4.792 eV, and the highest pixel distribution was observed ≈4.79 eV. These work function measurements of the bottom electrode indicates that the Cu (111) bottom electrode corresponds to the conditions for high‐quality SAMs and charge transfer properties. Also, the quality of SAM is intimately associated with the roughness of bottom electrode, bottom electrode roughness (Figure S7, Supporting Information) was probed with AFM topographical imaging. Cu (111) bottom electrode RMS roughness was distributed as 3.173 nm (2A5MT), 2.838 nm (3ATZ), 3.344 nm (PTT), 2.19 nm (REF). To clarify the distribution of chemisorption strength during self‐assembly, the adhesion force of SAM was spatially mapped through AFM FD curve measurements (Figure S8, Supporting Information). The adhesion force profile, which directly indicates the adsorption strength as the force required for desorption after contact of the Pt AFM tip with the SAMs surface, reveals that the SAM is stably chemisorbed, and the scale of the adhesion force is distributed in the typical chemisorption range.^[^ ^34^ ^]^

### 2.2 DFT Calculation of Atomic‐Scale Characteristics at Metal–Molecule Interfaces

**Figure 2:**
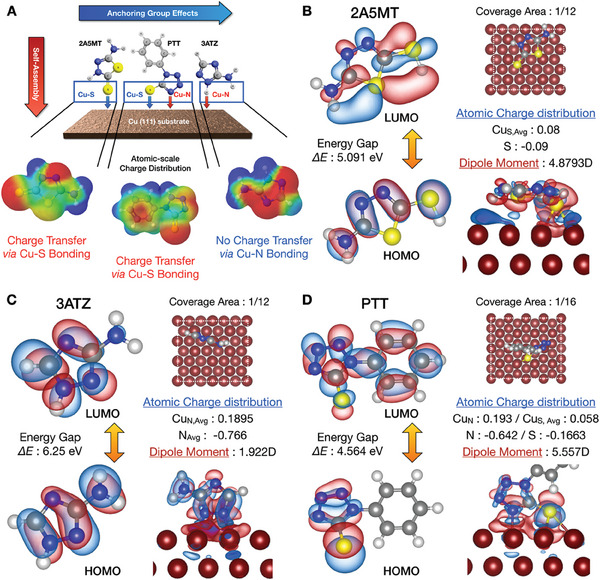
DFT‐calculated atomic‐scale characterization of metal‐molecule interfaces. A) Schematic of the self‐assembly process on the Cu (111) surface and the simulated atomic charge distribution. DFT‐calculated electrochemical properties of B) 2A5MT, C) 3ATZ, and D) PTT, including atomic charge distribution, HOMO‐LUMO gap, and dipole moment. The isosurface value for HOMO and LUMO is set at 0.03 e Bohr^−3^. DFT calculated atomic charge distribution theoretically correlates with the heterogeneous tunneling dynamics between Cu─S bonding and Cu─N bonding, which attributed to the interface dipole and electron push/pull effects. The isosurface value of differential charge distribution is 0.015 e Bohr^−3^.

While covalent bonding generated hybrid tunneling states at the metal–molecule interface during the self‐assembly process, as shown in **Figure** **2**A, the selected heterocyclic compounds established strong covalent bonds with the Cu (111) surface through electrochemical interactions between the lone electron pairs of the heteroatoms and the vacant d orbitals of the metal atoms.^[^ ^35^ ^]^ This orbital overlap facilitates efficient charge transfer via the formation of hybrid tunneling states between the anchored heteroatom and Cu atoms, thereby enabling effective charge transfer at the molecular junction.^[^ ^36^ ^]^

DFT calculations were performed for correlative matching of the experimental results and theoretical calculations to reliably validate the formation of hybrid tunneling states at the metal–molecule interfaces. The charge distribution values were plotted with the corresponding single‐molecule junctions to theoretically determine the electron pushing and pulling effects, which were dominantly governed by the atomic charge distribution. After constructing a single‐molecule junction, the heterocyclic compounds were deprotonated (Figure S9, Supporting Information) and adsorbed onto the Cu (111) surface, with the ring lying flat on the metal surface.^[^ ^37^ ^]^ This is distinct from the covalent bond information on an SAMs scale. However, even if the detailed covalent bond information differs, the charge distribution and charge transfer direction of each atomic variable are determined by the intrinsic atomistic properties. Thus, a flat heterocyclic ring on the metal surface can be theoretically ignored. When a molecule is chemisorbed onto a coinage metal, the broadening effect^[^ ^38^ ^]^ and image charge effect^[^ ^39^ ^]^ usually occur. However, DFT calculation of heterocyclic compounds itself can distinguish the atomic charge distribution difference between nitrogen and sulfur atoms. Also, since the atomic charges of nitrogen and sulfur are both negatively charged, HOMO‐LUMO shift by image charge effect and broadening effect occurs in the same direction, therefore, the image charge effect and broadening effect can be excluded from HOMO‐LUMO and energy diagram analysis.

The energy‐level diagram of the highest occupied molecular orbital (HOMO)–lowest unoccupied molecular orbital (LUMO) gap was constructed using the B3LYP method. As shown in Figure 2B,C, while the 3ATZ molecule anchored with a nitrogen‐anchoring group was characterized by the most substantial HOMO–LUMO energy gap (5.45 eV) the energy gap of 2A5MT with a sulfur‐anchoring group had a notably lower HOMO‐LUMO gap (4.06 eV). In Figure 2D, PTT anchored with sulfur and nitrogen atoms affirmed the existence of a 4.56 eV energy gap situated between those of 2A5MT and 3ATZ. These atomic characteristics establish that the nitrogen atom, which is endowed with a strong negative charge, hinders charge transfer through Cu─N bonding, even under a tip‐induced optoelectrical field. This was primarily due to the repulsive force exerted by the nitrogen atoms. Conversely, the sulfur atom, which has a comparatively low negative charge, permits electron migration from the metal surface to the molecule via Cu─S bonds under the influence of an optoelectrical field. In addition, Bader atomic charge analysis was performed on the optimized adsorption structural configuration to quantify the atomic charge distribution of the anchoring group and Cu atom, which manifested charge amounts of −0.09 and −0.1663 e^−^ for the sulfur atom of the Cu─S bond present in 2A5MT and PTT, respectively. On the contrary, the nitrogen atom of the Cu─N bond exhibited in 3ATZ and PTT showed charge distributions of −0.733 and −0.642 e^−^, respectively. At this time, a high charge distribution (−0.733 and −‐0.642 e^−^) and electronegativity (3.04) in the nitrogen atoms of the Cu─N bonds led to an electron‐pushing effect that repelled electrons. In contrast, for sulfur atoms, an electron‐pulling effect, which diffusively stabilized the thermodynamic states,^[^ ^40^ ^]^ was induced because of the relatively low charge distribution (−0.09 and −0.1663 e^−^) and electronegativity (2.58). The DFT calculations of the atomic charge distribution were precisely aligned with the observed intrinsic charge transfer properties, as shown in Figure 1B.

The DFT‐calculated HOMO‐LUMO gap of 3ATZ (6.25 eV) calculated by DFT was larger than those of PTT (4.564 eV) and 2A5MT (5.091 eV), and this heterogeneity in the HOMO‐LUMO gap resulted from the difference in electronegativity between nitrogen and sulfur Because a higher (lower) HOMO‐LUMO gap was associated with a higher (lower) atomic charge distribution, an electron‐pushing (pulling) effect was induced in the nitrogen (sulfur)‐anchored domain, which efficiently blocked (facilitated) the interfacial charge transfer through Cu−N (Cu−S) bonding. The metal Fermi level and HOMO‐LUMO levels were modulated by generating localized electrical and near fields at the tip–sample junction, (Figure S1**0**, Supporting Information). thereby enabling selective and remote interface engineering. Consequently, as the energy level shifted according to the direction of the electrical field, the heterogeneity of the charge transfer dynamics was clearly observed.

### 2.3 Polarization‐Dependent Interfacial Electron Tunneling Control of a Metal–Molecule Heterostructure

**Figure 3:**
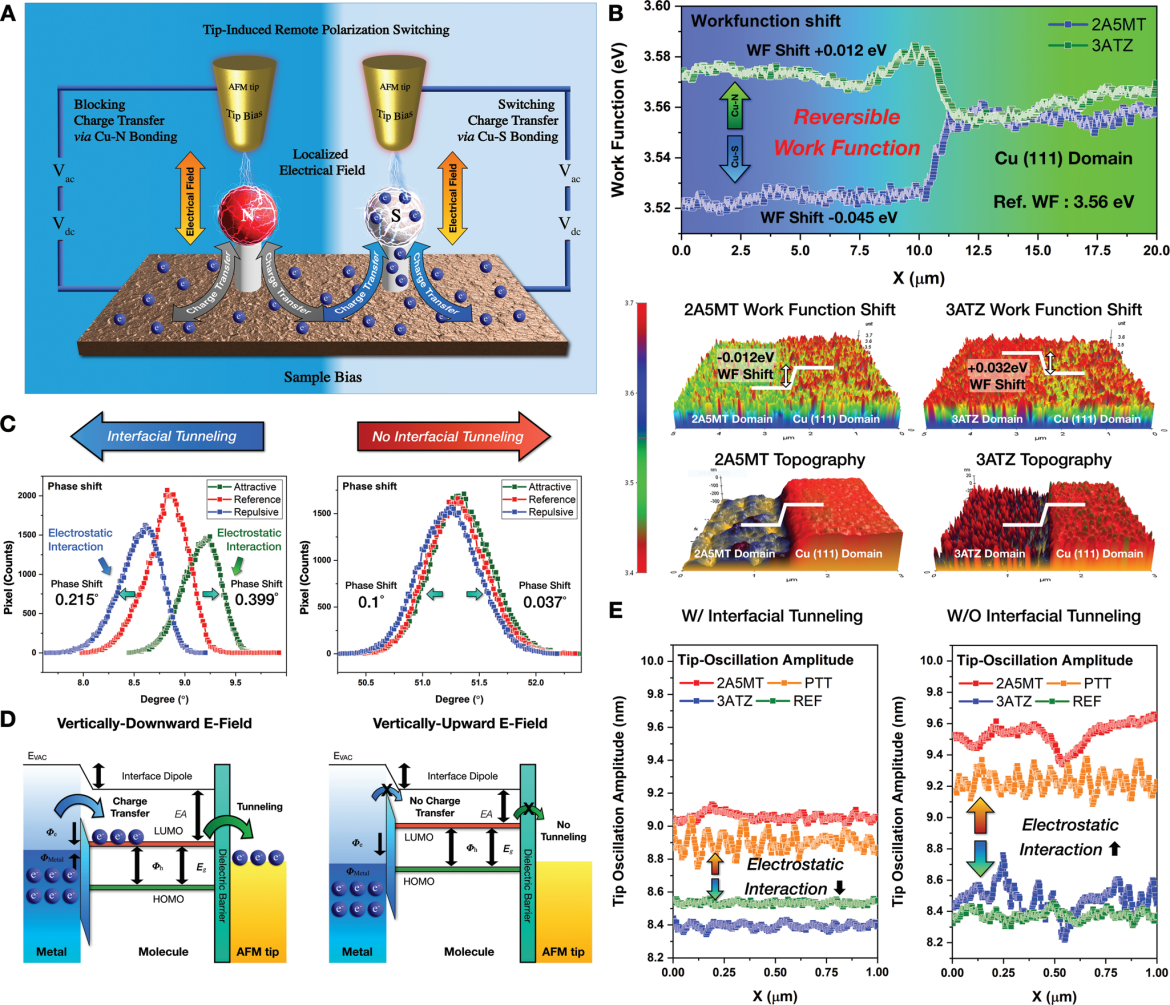
Polarization‐dependent heterogeneity of interfacial electron tunneling dynamics. A) Schematic illustration of KPFM tip‐induced polarization switching and polarization‐dependent charge transfer mechanism. Nanoscale heterogeneity in B) work function shift, C) phase shift, and E) tip oscillation amplitude, which dominantly attributed to polarization‐dependent electrostatic interaction and atomic charge distribution. D) Energy level diagram of metal (Cu)‐molecule‐dielectric barrier (air)‐AFM tip (Pt) junction, describing the mechanism that charge transfer to the AFM tip through the tunneling gap.

To investigate the heterogeneity of the interfacial tunneling dynamics originating from the electron pushing and pulling effects, the interfacial charge transfer was remotely controlled through tip‐induced polarization switching. As tip‐induced polarization switching generates a localized electric field at the tip–sample junction (**Figure** **3**A), the positive (negative) tip bias and negative (positive) sample bias establish a downward (upward) electric field. Because Cu−S bonding enables electrons to tunnel toward the SAMs (i.e., the electrical potential is lower than that of the Cu (111) surface), the sulfur atom facilitates the electron‐pulling effect, which efficiently activates interfacial tunneling under a downward electric field. Nitrogen atoms reversibly generate electron‐pushing effects that effectively inhibit interfacial tunneling.

By applying an AC modulation voltage and a DC bias voltage between the sample and tip for surface potential measurement, the electrostatic interaction at the tip‐sample junction was clearly detected by analyzing the work function, electrical potential, oscillation amplitude, and phase shift in a KPFM system based on a locking amplifier. Under a tip‐induced localized electrical field, the work function can be reversibly tuned according to the anchoring group, as shown in Figure 3B. Because the work function decreased in the absence of a tip‐induced localized electric field (Figure S2, Supporting Information), the charge transfer dynamics implied that the reversible work function shifted with the intratrmolecular charge density. Under a downward electric field, the intramolecular charge density of 2A5MT increased and the work function decreased by 0.045 eV compared with that of the Cu (111) surface, whereas in 3ATZ, the intramolecular charge density decreased and the work function increased by 0.012 eV. As the reversible work function shift is attributed to interfacial charge transfer, the work function decreased owing to increased repulsion between electrons as the charge density on the surface increased, owing to the interfacial charge transfer occurring through Cu−S bonding. However, the work function increased due to the low charge density at the Cu─N bond, which inhibits interfacial charge transfer. In addition, the observed reversible work function shift was further validated using electrical potential (Figures S11 and S12, Supporting Information). The electrical potentials were further measured under downward and upward electrical field conditions, where interfacial charge transfer occurred and did not occur, respectively. As the electrical potential and work function exhibited opposite tendencies, the work function shift was −0.021 eV for 2A5MT and −0.013 eV for 3ATZ under an upward electrical field (Figure S2, Supporting Information). While SAMs‐based tunneling junction was constructed and probed with KPFM and PiFM in the 5 µm × 5 µm region, 256 pixels × 256 pixels, three samples each, the statistical work function pixel distribution (Figure S13, Supporting Information) clarifies the interface tunneling occurrence. It indicates that 2A5MT and PTT, where interfacial tunneling occurs, decrease relative to the Cu (111) work function, and 3ATZ, where interfacial tunneling does not occur, increase relative to the Cu (111) work function. The Cu (111) region has a maximum pixel distribution at 3.56 eV, while 2A5MT has a maximum pixel distribution at 3.53 eV, PTT at 3.52 eV, and 3ATZ at 3.58 eV.

Under the upward electric field, the electrical potential shift was homogeneously shifted by +0.038 and +0.017 mV for 2A5MT and 3ATZ, respectively. Furthermore, an upward electric field with a negative AFM tip bias resulted in electrical potential shifts of +0.052 and +0.019 mV for 2A5MT and 3ATZ, respectively. In contrast, a downward electric field induced interfacial charge transfer, exhibiting reversible electrical potential shifts of −0.038 and +0.017 mV for 2A5MT and 3ATZ, respectively, which is correlated with the reversibly tunable work function shown in Figure 3B. Moreover, interfacial charge transfer induced by a positive AFM tip bias stimulated a potential shift due to interfacial charge transfer, resulting in inhomogeneous electrical potential shifts of −0.052 and +0.019 mV for 2A5MT and 3ATZ, respectively. Thus, although the potential distribution of each sample was similar in the upward electric field, potential heterogeneity among 3ATZ, REF, and 2A5MT was clearly observed in the downward electric field. Moreover, under an upward electric field, charge transfer was not facilitated by the electron‐repelling barrier of the nitrogen atom, which represents the electrical potential of each domain. In addition, tip–sample electrostatic interactions have been experimentally observed with tip‐oscillation amplitude modulation and phase shift under specific conditions such as sulfur anchoring and a downward electric field. As shown in Figure 3C, the sulfur‐anchored domain stimulates interfacial tunneling and enhances electrostatic interactions at the tip–sample junction, resulting in heterogeneous phase shifts of −0.215° / +0.399° (2A5MT) and −0.217° / +0.245° (PTT). Moreover, heterogeneous phase shift was further observed with positive AFM tip bias, as −0.915° / +0.336° (2A5MT) and −0.012° / +0.055° (3ATZ). In contrast, the nitrogen‐anchored domain effectively hindered the interfacial tunneling and tip–sample interactions, exhibiting negligible phase shifts of −0.1° / +0.037° (3ATZ) and −0.062° / +0.084° (Cu_2_O) (Figures S14 and S15, Supporting Information).

As shown in Figure 3D, the observed charge transfer dynamics can be correlated with the energy‐level alignment diagram. This correlation can be achieved by associating the HOMO‐LUMO gap and interfacial charge transfer with the diagram of the energy of the Fermi level in the metal and the LUMO of the molecule. If the Fermi level of Cu (111) is higher than the LUMO of the molecule, electrons in the metal are blocked from passing through the tunneling gap. Thus, the metal Fermi level and HOMO‐LUMO levels of the molecule can be shifted by applying a negative bias to the tip and a positive bias to the sample, thereby shifting the energy level depending on the direction of the electrical field. The heterogeneity of the interfacial charge transfer dynamics is clearly exhibited in the Cu─S and Cu─N bonds, according to the electron pushing and pulling effects.

Furthermore, the tip oscillation amplitudes exhibited tip–sample interactions, including electron pulling and pushing effects, as shown in Figure 3E. As the tip voltage is consistent, the electrical potential difference between the tip and samples increased from 1993.2 to 2042.7 mV owing to the potential of the sample due to interfacial charge transfer. Furthermore, because the tip oscillation amplitude is proportional to the potential difference between the tip and the sample,^[^ ^41^ ^]^ the tip oscillation amplitude increases significantly only in the sulfur‐anchored domain, which clearly indicates the heterogeneity of the charge transfer. Under the downward electrical field condition, the highest electrical potential difference in the sulfur‐anchored domain stimulated higher tip oscillation amplitudes of 9.51 nm (2A5MT) and 9.23 nm (PTT). Conversely, the nitrogen‐anchored domain exhibited low amplitudes of 8.55 nm (3ATZ) and 8.38 nm (REF). nm in the absence of an electric field, 2A5MT and 3ATZ exhibited comparable tip oscillation amplitudes of 9.07 and 8.47 nm, respectively.

To theoretically understand the electrostatic interaction at the tip—sample junction, tip–sample junction modeling was performed under a tip‐induced localized electrical field. Theoretical modeling was defined by parameters such as amplitude (*d*), spring constant (*c*), frequency (*f*_0_), and average tip‐molecule distance (*Z*). The molecule oscillates between polarized and nonpolarized states, directly indicating the oscillating motion of the tip. Despite the variation in the charge quantity owing to the tunneling process, the average charge aligns with the tip oscillation amplitude and imposes an oscillating electrostatic force on the cantilever tip. This leads to an observable shift in the cantilever resonance frequency, which is directly proportional to the electrostatic force *F*(*t*) applied to the AFM tip. Although the randomness of tunneling accelerates charge quantity fluctuations, the average value synchronizes with the oscillating movement of the tip, thereby applying an oscillating electrostatic force to the cantilever tip.

(1) $$\Delta f\;=\;-\frac{f_{0}^{2}}{cd}\int_{0}^{\frac{1}{f_{0}}}F\left(t\right)\cos\left(2\pi f_{0}t\right)\;dt$$

Electrostatic force *F*(*t*) induces an observable shift in the cantilever resonance frequency Δ*f*, as shown in Equation (1).^[^ ^42^ ^]^ Hence, the cantilever resonance frequency will shift proportionally to the electrostatic force *F*(*t*).^[^ ^43^ ^]^ Thus, the theoretical estimation that a positive (negative) *F*(*t*) corresponds to a negative (positive) shift in the cantilever resonance frequency Δ*f* indicates an exact agreement with Figure 1B,C.

### 2.4 Near‐Field Spatio‐Spectroscopic Investigation of Heterogeneous Interfacial Tunneling Dynamics

**Figure 4:**
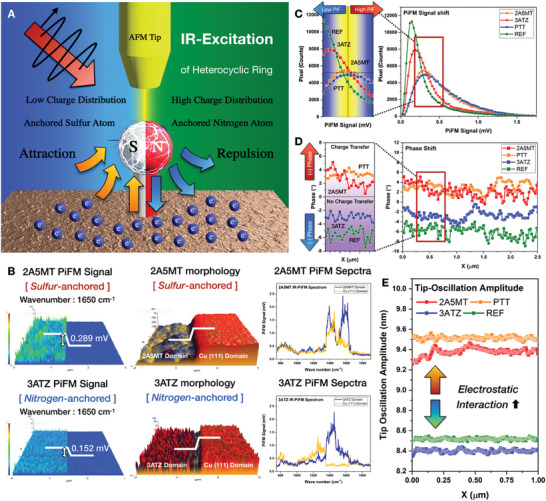
Near‐field spatio‐spectroscopic heterogeneity of interfacial tunneling dynamics. A) Schematic illustration of PiFM IR‐excitation switching and photo‐induced charge transfer mechanism. B) Nanoscale spatio‐spectroscopic mapping of morphologies, PiFM IR spectra, and photo‐induced force at the specific wavenumber, which exhibits the highest difference of optoelectrical response. Multidimensional near‐field heterogeneity of C) PiFM signal intensity shift and D) phase shift, E) tip oscillation amplitude, which is accumulatively induced by near‐field electrostatic interaction and photo‐induced charge transfer.

To further spatio‐spectrally demonstrate the electron tunneling behavior, a systematic investigation was performed via hyperspectral PiFM imaging, as illustrated in **Figure** **4**A. When laser excitation generated a tip‐enhanced localized near‐field at the tip–sample junction, photoinduced interfacial charge transfer was facilitated through the hybrid tunneling states. Using tip‐induced excitation switching, interfacial charge transfer was spatio‐spectrally observed in non‐destructive states depending on the heterogeneous charge density of the anchored heteroatoms. According to Figure 4B,C, infrared (IR) excitation wavenumber sweeping in the quantum cascade laser (QCL) sweep range (750–1800 cm^−1^) was proactively conducted to determinate the highest spectral PiF peak intensity difference at the PiFM IR‐spectra. The PiFM IR absorption spectra revealed that each self‐assembled heterocyclic ring exhibited distinguishable vibrational resonances, leading to amplified PiFM signals at specific wavenumbers. As shown in Figure 4D, the maximum PiFM signals of 3ATZ and 2A5MT were observed at 1650 cm⁻¹ and corresponded to C═N bonding within the triazole and thiadiazole rings. PTT exhibited the maximum PiFM signal at 1480 cm⁻¹, which was associated with the C═C bonding in the benzene ring. In contrast, the reference sample showed homogeneity in the PiF response, which was attributed to the absence of the corresponding chemical bonding in the QCL sweeping range.

By correlating the IR spectrum with the chemical structure, the IR resonance wavenumbers of each heterocyclic ring were selectively identified, thereby enabling a correlation between the intramolecular charge density and PiFM signal. A spatial PiFM signal shift of 0.289 mV was obtained for 2A5MT, which is a sulfur‐anchored domain, whereas 3ATZ, which is anchored to nitrogen, exhibited a lower PiFM signal shift of 0.152 mV. The observed PiFM spatiospectral heterogeneity aligns precisely with the tunneling‐dominant charge transfer dynamics, which were previously demonstrated through tip‐induced polarization switching. At a shifted wavenumber (1000 cm^−1^), the heterogeneous PiF signal exhibited a relatively low IR response as a function of the wavenumber and covalent bonding (Figure S16, Supporting Information). Due to the IR excitation switching, 2A5MT shifted from 0.147 mV (1000 cm^−1^) to 0.441 mV (1650 cm^−1^), 3ATZ shifted from 0.159 mV (1000 cm^−1^) to 0.313 mV (1650 cm^−1^), and PTT shifted from 0.151 mV (1000 cm^−1^) to 0.401 mV (1480 cm^−1^). Because the homogeneity of PiF at 1000 cm^−1^ precisely matched the IR‐PiFM spectra, PiF reactivity, which indicates photo‐induced charge transfer, was spatially observed with IR‐excitation switching. Moreover, the spatial PiF heterogeneity was correlated with the dipole moment and sample polarizability because the PiF was due to induced dipole–dipole interactions and tip‐enhanced thermal expansion. Although the dipole moment and sample polarizability exhibited a proportional correlation with the induced dipole–dipole interaction force, interfacial electron tunneling can be indirectly observed with spatial PiF heterogeneity. Since near‐field tip‐sample interactions exhibit a significant correlation with the occurrence of interfacial charge transfer, tip–sample interactions were investigated through phase shifts and AFM tip amplitude modulations acquired concurrently with sub‐10 nm resolution optoelectrical imaging (Figure S17, Supporting Information). As shown in Figure 1B,C, the negative (positive) phase shift was attributed to the repulsive (attractive) force of the AFM tip. This repulsive (attractive) force facilitated a decrease (increase) in the tip resonance frequency, thereby inducing a negative (positive) phase shift in the dynamic system. As shown in Figure 4D, the sulfur‐anchored domain, where interfacial tunneling occurred, exhibited positve phases of 2A5MT (+3.721°) and PTT (+4.192°), corresponding to an attractive force. Conversely, the nitrogen‐anchored domain exhibited negative phases of 3ATZ (–3.164°) and REF (–5.931°), which originated from repulsive forces.

In addition, Figure 4E shows the tip oscillation amplitudes, which directly indicate tip–sample interactions. The heterogeneity of the tip oscillation amplitudes at the tip–sample junction was dependent on the presence or absence of interfacial charge transfer within the tip‐enhanced near field. For the sulfur‐anchored domain, where intramolecular charge transfer occurred, the tip oscillation amplitudes were 9.34 nm (2A5MT) and 9.53 nm (PTT). Conversely, the tip oscillation amplitudes of the nitrogen‐anchored domain and reference with no charge transfer were 8.39 nm (3ATZ) and 8.52 nm (REF). Despite similar tendencies in the tip oscillation amplitudes for both tip‐induced localized electrical fields, the relative amplitude difference was suppressed, compared to that in the absence of a tip‐induced optoelectrical field. To theoretically clarify the optoelectrical origin of the heterogeneous PiF response, a PiF profile was modeled based on the sum of the induced dipole forces and tip‐enhanced thermal expansion forces. Under IR excitation, the induced dipole force was symmetrically generated by the dipoles at the tip apex and the sample, which actively interacted with each other. The Coulombic force in the tip‐enhanced near field is defined as the interaction between the induced dipoles of the tip apex and the sample, and it is modeled using Equation (2):

(2) Fdipz≈−3Reαt∗αs2πε02z4Ei2

where *α_t_* and *α_s_* are the tip and sample polarizabilities, respectively, *z* is the distance between the center of the tip apex dipole and the surface, and *E_i_* is the incident field. In other words, the photo‐induced charge transfer due to IR excitation determines the intramolecular charge distribution, which in turn determines the sample polarizability. According to Equation (2), the sample polarizability of the PiF is determined by the induced dipole force, which affects the interfacial charge transfer. In addition, because the tip‐enhanced near‐field also generates a tip‐enhanced thermal expansion force at the tip–sample junction, the thermal expansion (Δ*L*(*z*)) coupled with the tip–sample intermolecular force (*F_ts_*) gradient is defined by Equation (3):

(3) $$F_{\mathrm{th}}\left(z\right)\approx -\frac{\partial F_{ts}}{\partial z}\sigma d\Delta T$$

where σ is the coefficient of linear thermal expansion, *d* is the sample thickness, and Δ*T* is the temperature increase. Similarly, a tip‐enhanced thermal expansion force was induced through IR excitation, concurrently inducing an increase in the intramolecular charge distribution and tip–sample intermolecular force (*F_ts_*). In other words, the *F_ts_* gradient and PiF were proportional, enabling heterogeneous thermal expansion forces to facilitate interfacial charge transfer. In addition, a near‐field tip–sample interaction model was theoretically constructed based on various parameters including the tip oscillation amplitude (*A*), frequency shift (Δ*f*), and excitation frequency (*f_m_*).^[^ ^44^ ^]^ An excitation‐induced near field typically amplifies the tip–sample electrostatic interaction, including the phase and tip oscillation amplitudes. In this situation, α_*te*_ is the effective polarizability of the tip; α_*t*_ and α_*s*_ are the polarizabilities of the tip and sample, respectively; *d* is the average tip–sample distance; *a_t_* is the tip radius; and *E_i_* is the incident field. The *z*‐component of the electric field *E_tz_* acting on the tip can be expressed using Equation (4).

(4) $$E_{tz}=\left(1+\frac{\alpha_{s}}{2\pi {\left(Z+2a_{t}\right)}^{3}}\right)\;E_{i}$$

where *E_tz_* is proportional to the sample polarizabilities α_*s*_ because of the constant nature of *d*, *a_t_*, and *E_i_*. This implies that a localized electrical field enhances interfacial tunneling from the Cu (111) surface to the SAMs, subsequently enhancing sample polarizability α_*s*_. As the interfacial charge transfer occurred in sulfur‐anchored domains, the tip–sample junction was influenced by the electrostatic force. However, in nitrogen‐anchored domains, the absence of interfacial tunneling led to α_*s*_ becoming constant, which caused the tip to be unaffected by the electrostatic force. When interfacial tunneling occurs under a tip‐induced near field, the increase in *E_tz_* can become proportional to α_*s*_ and induce atomic‐scale electrostatic interactions on the AFM tip. Under a tip‐enhanced near field, a positive (negative) *F*(*t*) results in a negative (positive) shift in the cantilever resonance frequency Δ*f*, as shown in Equation (1). In addition, opto‐mechanical damping occurs owing to the influence of the tip‐induced near field.^[^ ^44^ ^]^

### 2.5 Tip‐Induced Tunneling Control at 4‐Inch Wafer‐Scale Vertical MIM Capacitor

**Figure 5:**
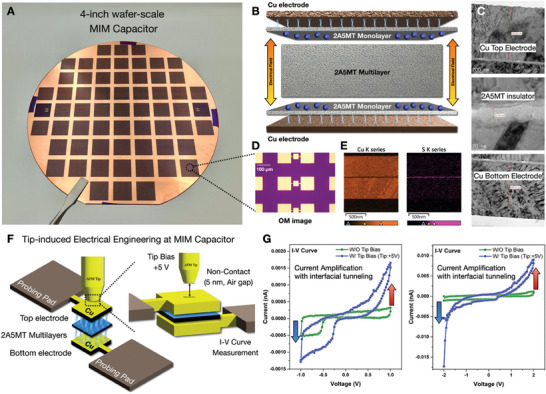
Tip‐induced tunneling control of 4‐inch wafer‐scale vertical MIM capacitor. A) Photography of fabricated 4‐inch wafer‐scale MIM capacitor. B) Schematic diagram of MIM capacitor structure, consist of metal electrode (Cu)‐insulator (2A5MT)‐metal electrode (Cu), enabling tunable capacitance based on 2A5MT monolayers. C) Cross‐sectional HR‐TEM image, D) OM image, and E) corresponding EDS elemental mapping of vertical MIM capacitor (Cu‐2A5MT‐Cu). F) Schematic illustration of remote tunnelling control platform through Cu─S bonding at Cu‐2A5MT‐Cu heterointerfaces. G) CV curve of different voltage sweep range, scanning the −1–+1 V (left) range and −2–+2 V (right) range. Tip‐induced electrical field remarkably enlarges the hysteresis, which is attributed to interfacial electron tunneling to the 2A5MT monolayer.

For the device‐scale demonstration of remotely controlled interfacial charge transfer, a 4‐inch wafer‐scale vertical metal–insulator–metal (MIM) capacitor was fabricated and characterized, as shown in **Figure** **5**A. As shown in Figure 5B, MIM capacitors were fabricated with a Cu–2A5MT–Cu structure. As the 2A5MT layer has tunable electrical properties depending on the dimension scale, 2A5MT multilayers were selected as the insulating layers of the MIM capacitor. Although charge transfer occurred efficiently in the 2A5MT monolayer through Cu─S bonding, the 2A5MT multilayer exhibited insulating properties, which were induced by intramolecular pushing effects in the N─N bonding (Figure S18, Supporting Information). Transmission electron microscopy (TEM) (Figure 5C) and optical microscopy (OM) (Figure 5D) were performed to correlate the top‐view and cross‐sectional images of the fabricated MIM capacitor. Based on TEM observation, the obtained thickness of each layer was 523.23 nm for the Cu bottom electrode, 25.39 nm for the 2A5MT insulator, and 539.01 nm for the Cu bottom electrode, respectively. Moreover, the corresponding energy dispersive spectroscopy (EDS) element mapping (Figure 5E) was conducted to further match the stable MIM structural construction of the Cu electrodes and 2A5MT insulator, based on the elemental distribution of Cu atoms (orange) and S atoms (magenta).

Cyclic voltammetry (CV) evaluations were performed within the tip‐induced localized electric field to investigate the effects of interfacial tunneling, as shown in Figure 5F,G. While the CV curves of the Cu–2A5MT–Cu capacitor exhibited hysteresis owing to electron tunneling across the 2A5MT insulator, the tip‐induced electrical field further enhanced the hysteresis. The interfacial tunneling was evidently observed at device‐scale via concurrent IV curve measurement with external tip bias (+5 V), while maintaining the tunneling gap (air, 5 nm) between the tip apex and top electrode. With external tip‐induced electrical field, a maximum current was measured at the +1 V with 2.485‐fold amplification (Figure 5G), and similarly at the +2 V with 5.211‐fold amplification (Figure 5H). The observed tunable hysteresis performance of the Cu–2A5MT–Cu capacitor can be attributed to the interfacial charge transfer through Cu─S bonding. Charge‐collecting effects in the 2A5MT monolayers, which are attributed to electron tunneling to the 2A5MT monolayers through Cu─S bonding, imply an equivalent effect of decreased insulator thickness and increased electrode thickness. Consequently, tunneling‐induced insulator‐thinning effects efficiently facilitate current amplification, which is in exact accordance with the previously demonstrated charge transfer dynamics shown in Figures 1, 2, 3, 4.

## 3 Conclusion

In summary, the remote control of interfacial tunneling dynamics in SAM‐based tunneling junctions was demonstrated via tip‐induced optoelectrical engineering. The heterogeneous interfacial charge transfer dynamics were governed by the charge density of the anchoring group and led to a work function shift in a reversible (up to 0.012 eV) fashion in sulfur‐anchored domains and an irreversible (up to −0.045 eV) fashion in nitrogen‐anchored domains. Tunneling‐based interfacial charge transfer dynamics can be understood in an optoelectrical way that has not been previously achieved through spatial mapping of the optical, electrical, and morphological properties of SAMs‐based tunneling junctions. The observed heterogeneous interfacial electron tunneling dynamics were theoretically supported by DFT simulations, which accurately aligned with the electron‐pushing and electron‐pulling effects. For a practical device‐scale demonstration, interfacial tunneling was remotely controlled with a tip‐induced electrical field in a 4‐inch wafer‐scale MIM capacitor, which exhibited a 5.211‐fold current amplification under the tip‐induced electrical field. As the remote controllability of interfacial charge dynamics is highly desirable, tip‐induced optoelectrical engineering will lead to versatile advances in molecular‐based optoelectronic devices. Thus, this approach provides an on‐demand optoelectrical remote control of interfacial charge transfer, which enables the non‐destructive spatial investigation of advanced molecular electronic devices.

## 4 Experimental Section

### Self‐Assembled Monolayer Preparation

A 4 cm × 4 cm coupon wafer comprising Ti 500 Å/Cu 7000 Å with Cu (111) was used in the experiments. The wafer was then treated with HF and rinsed with isopropyl alcohol to remove the native oxide layer and organic contaminants. The bare copper wafer was then immersed in an etchant solution for 1 min. The etchant solution consisted of deionized water (483.4 g), the complex agent [glycine] (5 g), pH adjuster [HNO_3_] (0.1 mL), oxidant [H_2_O_2_] (16.67 g), and heterocyclic compounds (0.25 g), namely 1) 2‐amino‐5‐mercapto‐1,3,4‐thiadiazole, 2) 3‐amino‐1,2,4‐triazole, and 3) 1‐phenyl‐1H‐tetrazole‐5‐thiol sodium salt. During etching, the self‐assembly proceeded concurrently to generate the SAMs directly on the Cu (111) surface.

### X‐Ray Photoelectron Spectroscopy Measurements and Data Analysis

XPS measurements (NEXSA, ThermoFisher Scientific, USA) were performed using an X‐ray spot size of 400 µm. Peak analyses were conducted on the C 1s, N 1s, S 2p, and Cu 2p spectra. The XPS profiles were aligned by correcting the C 1s peak to a binding energy of 285 eV. After the measurements, the data were calibrated using CASAXPS software (version 8.1).^[^ ^45^ ^]^

### Atomic Force Microscopy Measurements

KPFM (AFM; NX‐10, Park Systems, Republic of Korea) was conducted using an ElectriMulti75‐G cantilever (KPFM). The KPFM reference work function was set to the highly oriented pyrolytic graphite sample (4.76 eV). Because the KPFM silver paste electrode was attached between the sample plate and the Cu (111) wafer, charge transfer was only allowed at the metal–molecule interfaces. In addition, PiFM (NX‐IR, Park Systems, Republic of Korea) with a PPP‐NCHR cantilever was employed for the PiFM measurements. The QCL laser used for PiFM was adjusted to an intensity of 1%. Prior to the PiFM measurements, the QCL laser was focused on the initial spatial intensity positions directly under the AFM tip.

### Density Functional Theory Calculation

DFT calculations were conducted for all periodic systems using the Quantum Espresso package to investigate the work function and optimize the spatial structures. The exchange and correlation energy were treated using the Perdew–Burke–Ernzerhof function of the generalized gradient approximation. The projector‐augmented wave pseudopotential describes the ion‐electron interaction. The semi‐empirical DFT‐D3 method was adopted for the van der Waals dispersion terms. A Γ‐centered 4 ´ 3 ´ 1 Monkhorst–Pack k mesh was set for sampling the Brillouin zone. The supercells consisted of four Cu (111) surface layers, an adsorbed molecule, and a vacuum region with a thickness of >20 Å. For structural relaxation, the undermost Cu layer was fixed to represent the bulk state, while the other layers and atoms were fully relaxed until the Hellmann–Feynman forces were <0.002 Ry/Bohr and the energy convergence threshold was lower than 0.0004 Ry. The energy cutoff for the plane‐wave expansion was 60 Ry for all calculations. The electrostatic potential along the z‐direction was obtained using a dipole correction to offset the intrinsic electric polarization. The work function (W) of the entire system is the energy required to remove electrons from the surface under a vacuum. This is defined as Equation (5):

(5) $$W\;=V_{vacuum}\;-E_{F}$$

where *V_vacuum_* is the electrostatic potential at the vacuum level and *E_F_* is the Fermi energy. The amount of charge transferred between the adsorbed molecule and Cu (111) surface was determined using the Bader charge analysis. In addition, density functional theory (DFT) calculations of the three molecules under nonperiodic conditions were implemented in Python‐based simulations of the PySCF package. Geometric optimization and total energy calculations were conducted by applying Becke's three‐parameter mixed exchange function (B3) and the Lee–Chang Parr correlation function. Energy‐level orbital diagrams and the corresponding canonical molecular orbitals were calculated using the cc‐pVTZ basis set.

## Conflict of Interest

The authors declare no conflict of interest.

## Author Contributions

J.L., E.K., and J.C. contributed equally to this study. J.L. and E.K. prepared the samples and performed the AFM experiments. J.C. performed theoretical DFT calculations and modeling. H.S., G.W., and D.Y. performed technical discussions and measurements of the MIM capacitor devices. G.J., B.H., J.N., and I.I. conducted PiFM experiments. J.L., E.K., J.C., H.S., and G.W. analyzed the data and all authors discussed the results. J.L., E.K., and J.C. wrote the manuscript with contributions from all authors. T.K. designed and supervised the study.

## Supporting information

Supporting Information

## Data Availability

The data that support the findings of this study are available from the corresponding author upon reasonable request.
