# Supplementary material for: Remote‐Controllable Interfacial Electron Tunneling at Heterogeneous Molecular Junctions via Tip‐Induced Optoelectrical Engineering
Source: Adv Sci (Weinh). 2023 Dec 6;11(5):2305512. doi: 10.1002/advs.202305512 (PMC10837351; doi:10.1002/advs.202305512)
Supplement: Supplementary file 1 — Supporting Information [file ADVS-11-2305512-s001.pdf]

ADVANCED SCIENCE
Open Access

Supporting Information

for *Adv. Sci.*, DOI 10.1002/advs.202305512

Remote-Controllable Interfacial Electron Tunneling at Heterogeneous Molecular Junctions via Tip-Induced Optoelectrical Engineering

*Jinhyoung Lee, Eungchul Kim, Jinill Cho, Hyunho Seok, Gunhoo Woo, Dayoung Yu, Gooeun Jung, Hyeon Hwangbo, Jinyoung Na, Inseob Im and Taesung Kim**

Supporting Information

Remote-Controllable Interfacial Electron Tunneling at Heterogeneous Molecular Junctions *via* Tip-Induced Optoelectrical Engineering

*Jinhyoung Lee‡, Eungchul Kim‡, Jinill Cho‡, Hyunho Seok, Gunhoo Woo, Dayoung Yu, Gooeun Jung, Hyeon Hwangbo, Jinyoung Na, Inseob Im, and Taesung Kim**

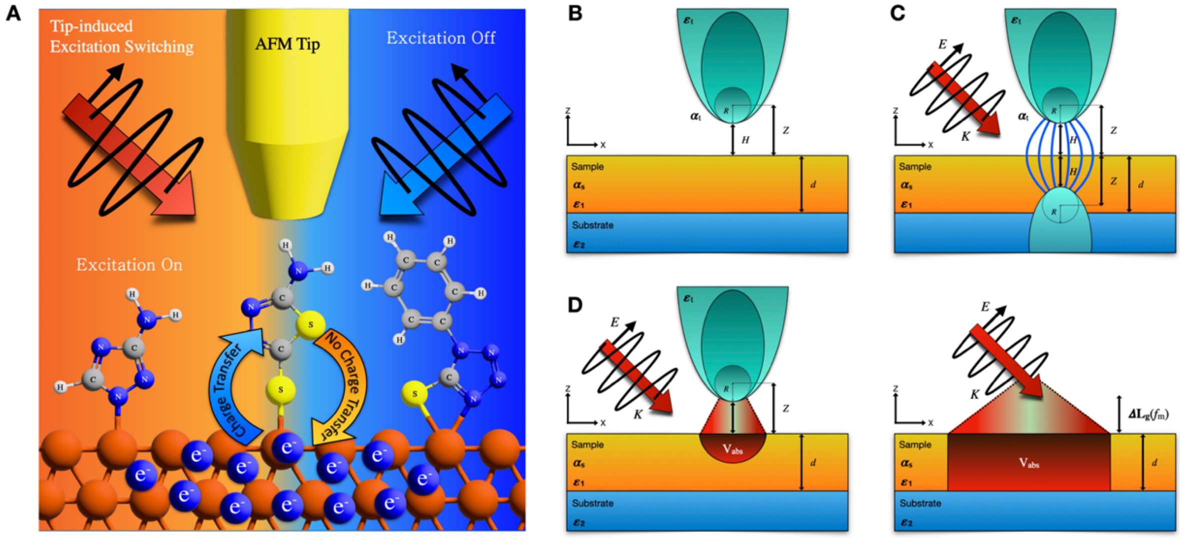

Figure S1. Mechanism of PiFM-based IR-excitation switching at the molecule junction systems, which efficiently induce the interfacial electron tunneling. Schematic illustration of a) before IR-excitation, b) induced dipole-dipole interaction, and c) tip-enhanced thermal expansion (left), enabling localized thermal expansion at the tip-sample junction, compared to global thermal expansion. (right)

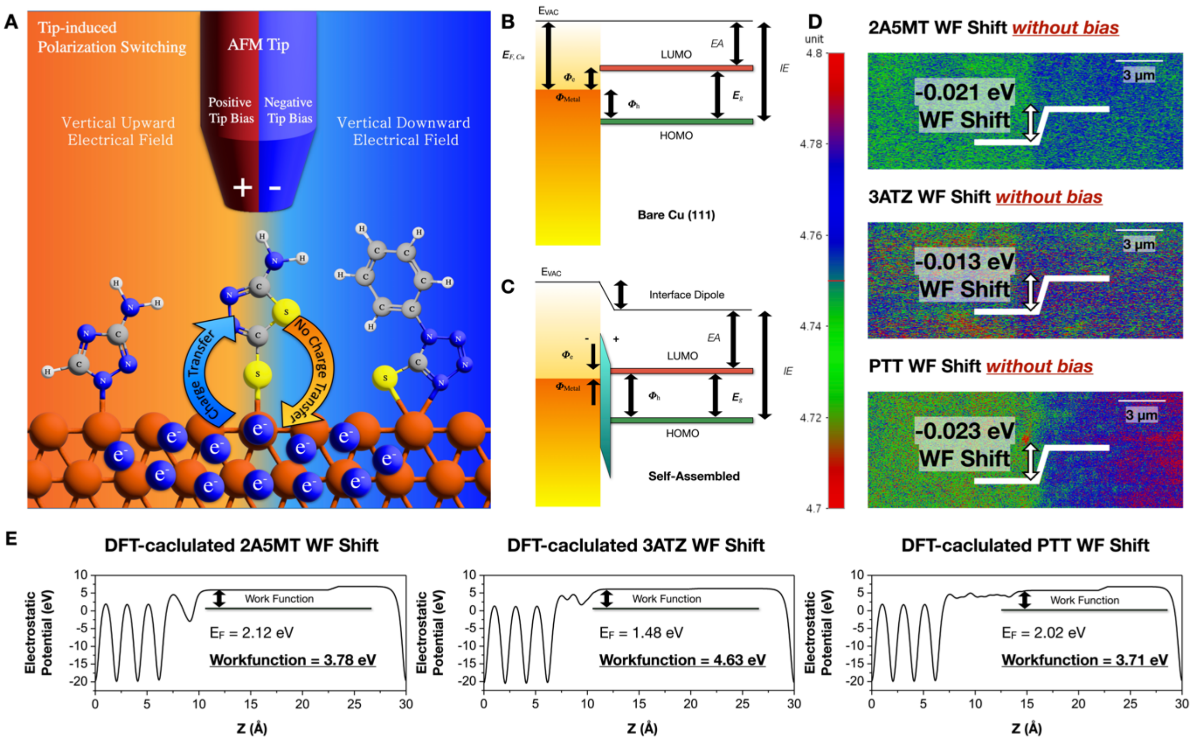

Figure S2. a) Mechanism of KPFM-based polarization switching at the molecule junction systems, which efficiently induce the interfacial electron tunneling. Energy level diagram of b) bare Cu (111) and c) self-assembled domain. In self-assembled domain, reduction of HOMO-LUMO level and work function corresponds with the interface dipole at the metal-molecule interfaces, thereby reducing the electron injection barrier. d) Spatial imaging of work function distribution at 2A5MT, 3ATZ, and PTT without biasing at tip-sample junction. e) DFT-calculated work function shift of SAMs, which correlatively match with HOMO-LUMO gap and KPFM work function measurements without external bias.

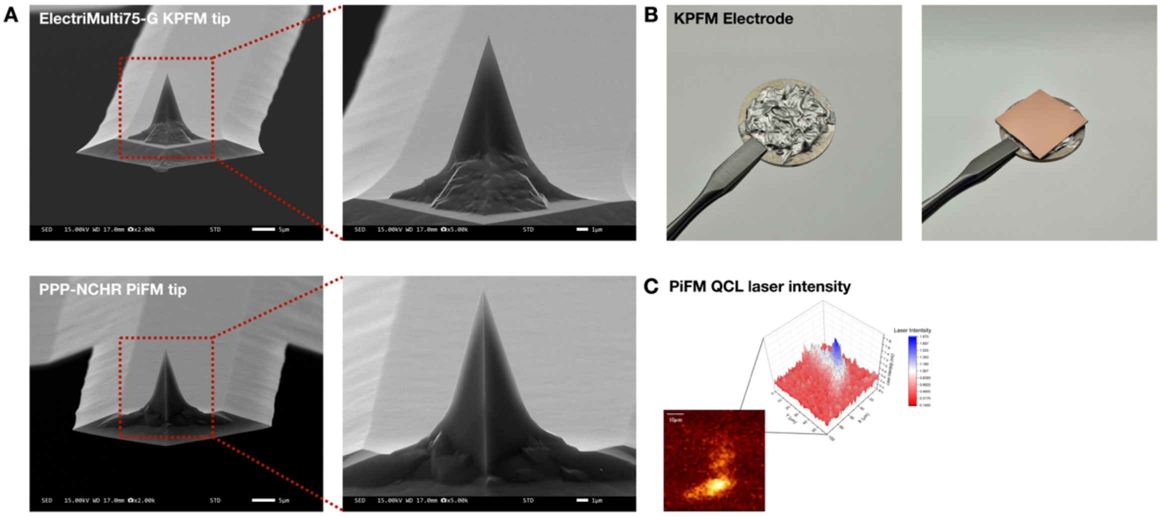

Figure S3. Experimental setup. a) FESEM image of KPFM ElectriMulti75-G tip (top) and PiFM NCHR tip (bottom) after all experiments. b) photography of KPFM sliver paste electrode (left) and attached Cu (111) wafer on the top of the sliver paste electrode (right), where interfacial charge transfer is the only possible. c) PiFM QCL laser intensity spatial map of initial focus positions.

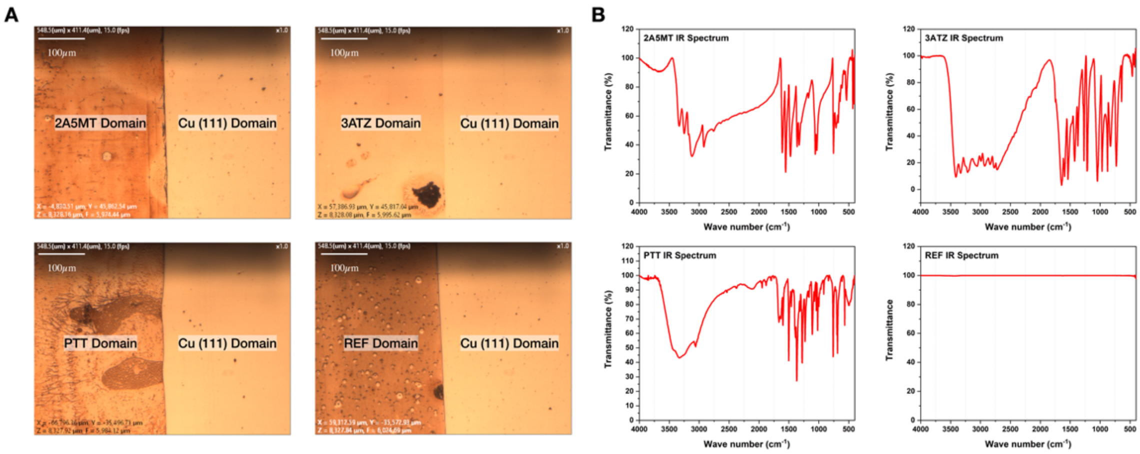

Figure S4. a) Optical heterogeneity between self-assembled domain and bare Cu (111) domain. b) FT-IR spectra of heterocyclic compounds, exhibiting the intrinsic chemical bonding compositions.

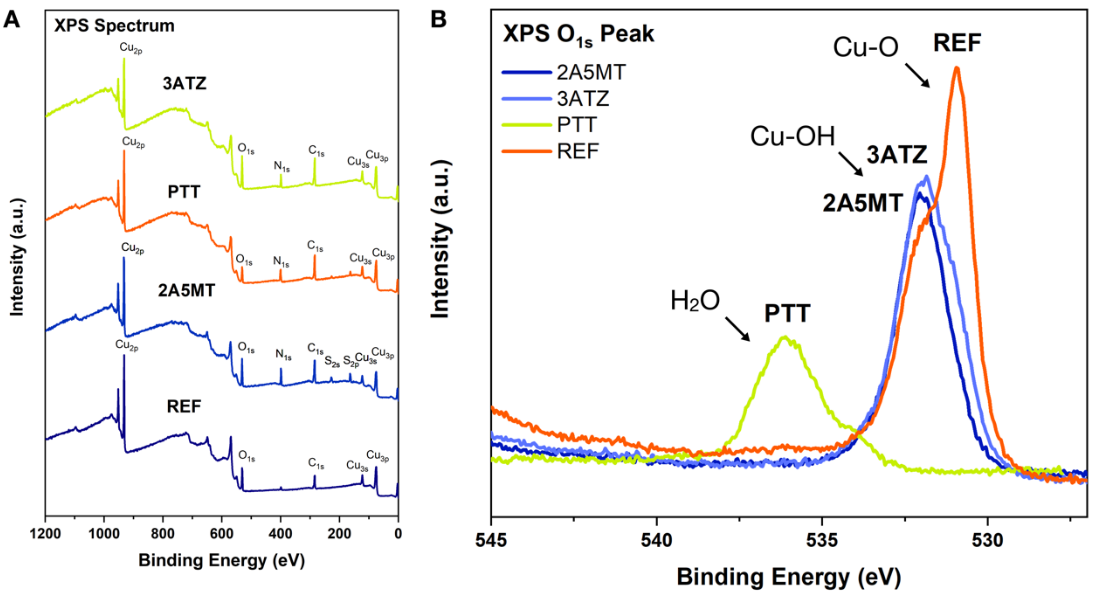

Figure S5. XPS spectra of organic self-assembled monolayers. a) XPS full spectrum, b) High-resolution XPS spectra of O 1s peak, which indicates surface oxidation inhibition *via* self-assembly.

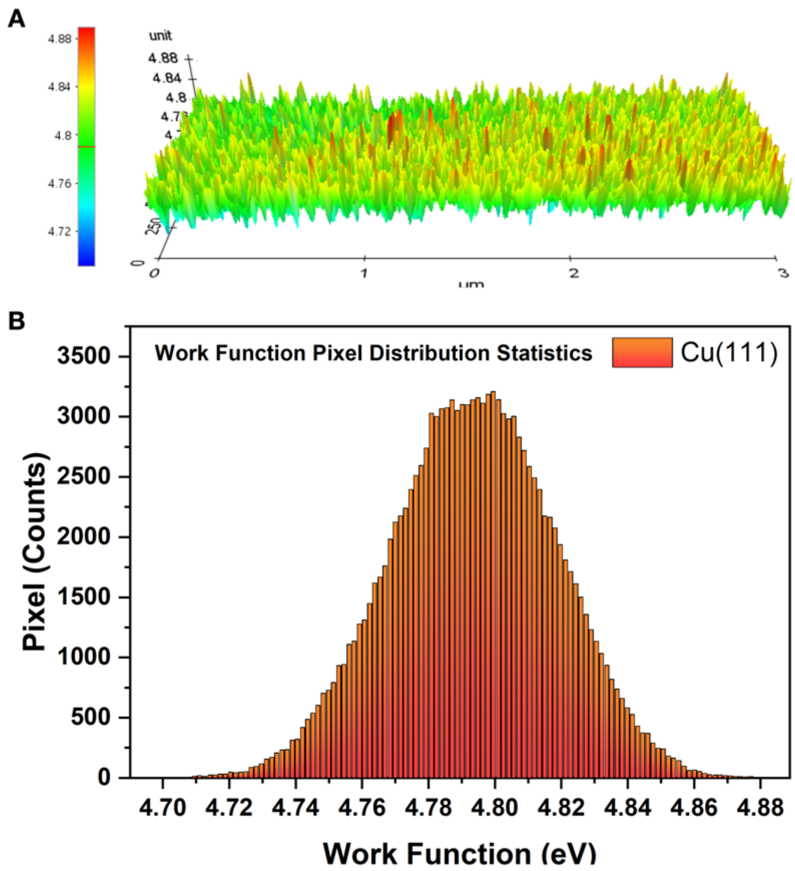

Figure S6. Work function measurement of Cu (111) electrode. a) KPFM work function 3D image, b) Work function pixel distribution statistics of Cu (111) electrode.

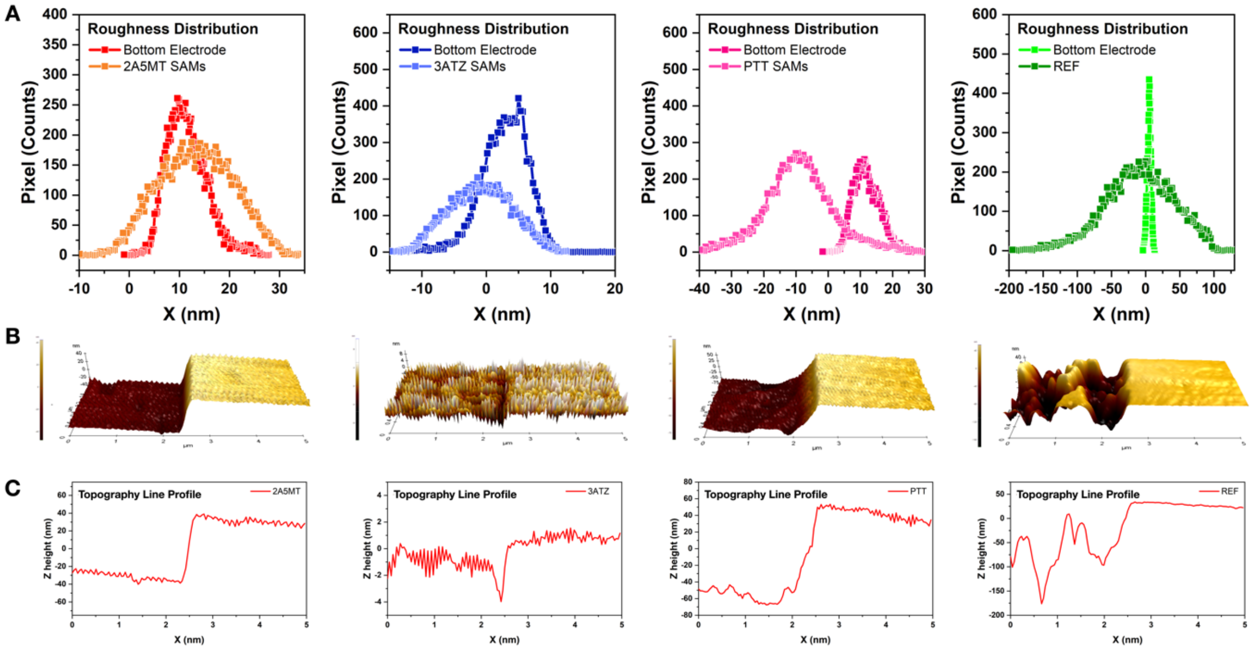

Figure S7. Surface roughness distribution of bottom electrode and SAMs. a) Surface roughness pixel distribution shift, b) 3D topography image, c) Z height profile of bottom Cu (111) electrode and each SAMs.

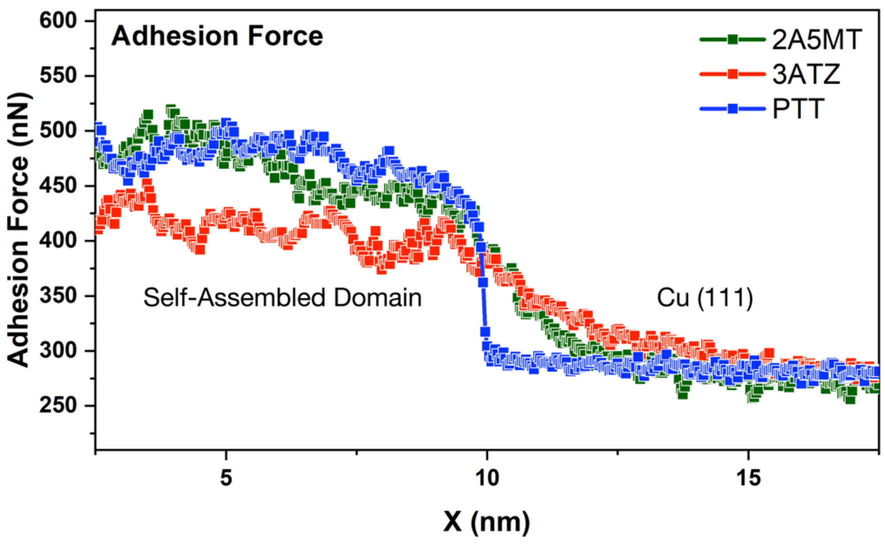

Figure S8. Adhesion force distribution between Cu (111) and SAMs domains, which indicates the larger adhesion force of the self-assembled domains compared to Cu (111).

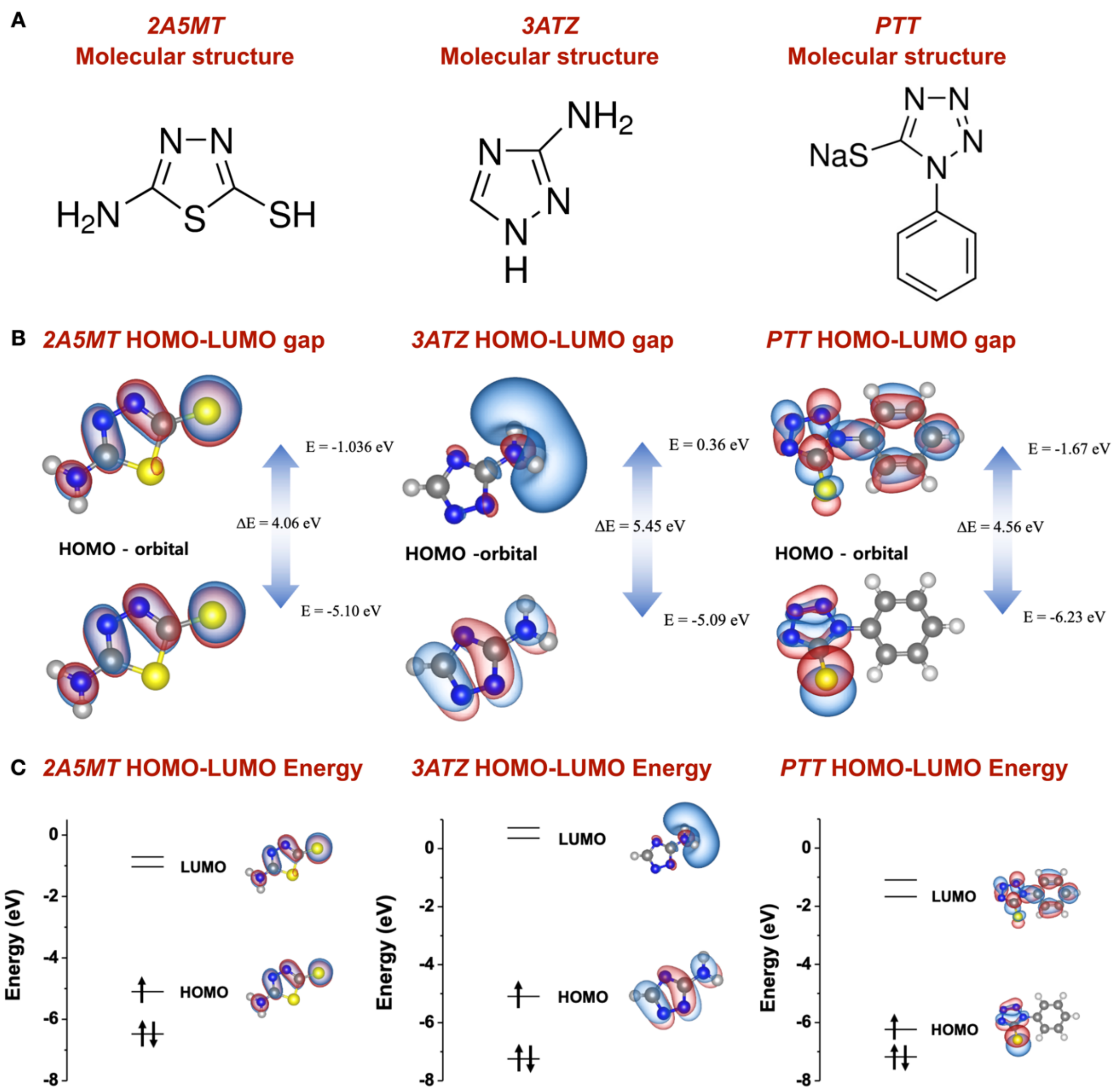

Figure S9. DFT calculation of deprotonated heterocyclic compounds. a) Molecular structure of 2A5MT, 3ATZ, and PTT. b) HOMO-LUMO energy gap and c) HOMO-LUMO energy level diagram of deprotonated 2A5MT, 3ATZ, and PTT.

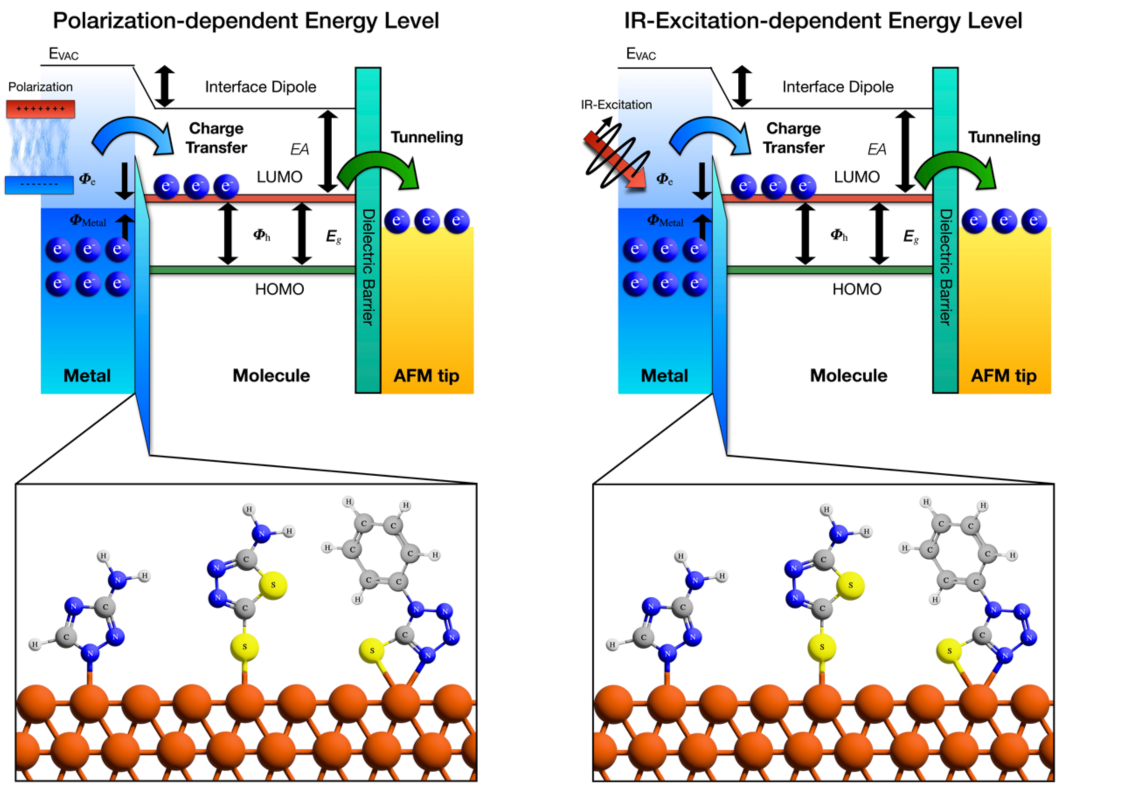

Figure S10. Energy level diagram of metal (Cu)-molecule-dielectric barrier (air)-AFM tip (Pt) junction with polarization (right) and IR-excitation (left), which generate the localized electrical field and near field at tip-sample junction, thereby enabling the remote-controllable interfacial charge transfer dynamics.

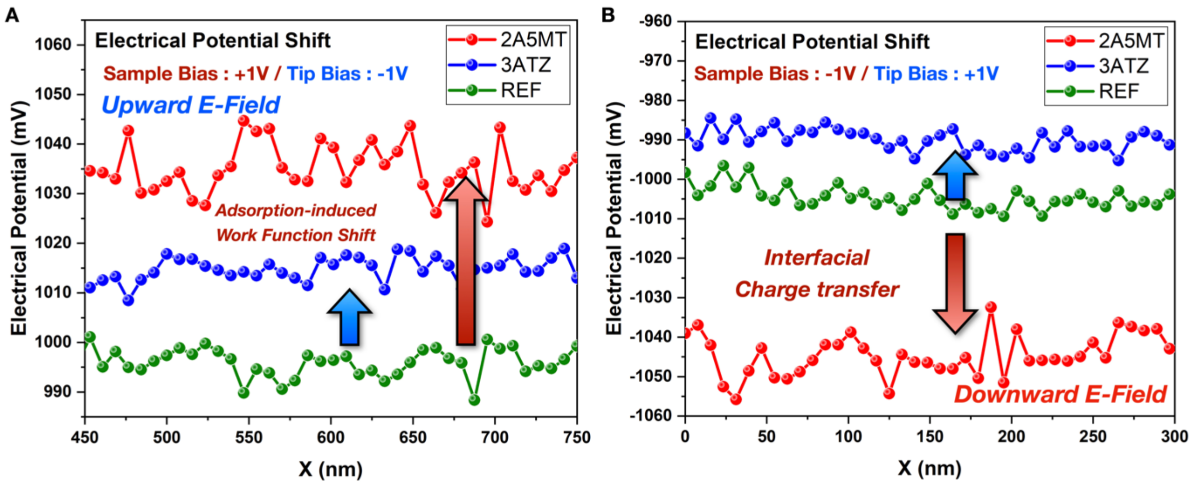

Figure S11. Electrical potential shift within a) upward electrical field and b) downward electrical field, which clearly exhibits the nanoscale heterogeneity of the sample polarizability, charge transfer dynamics and tip-sample electrical potential difference.

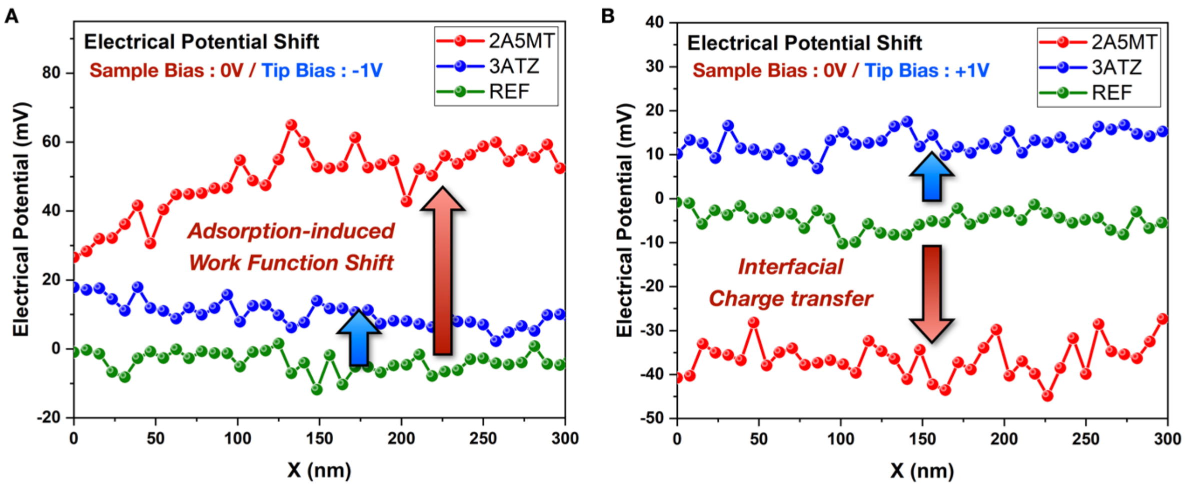

Figure S12. Tip bias effects of electrical potential shift within a) upward electrical field and b) downward electrical field, which clearly exhibits the nanoscale heterogeneity of the sample polarizability, charge transfer dynamics and tip-sample electrical potential difference.

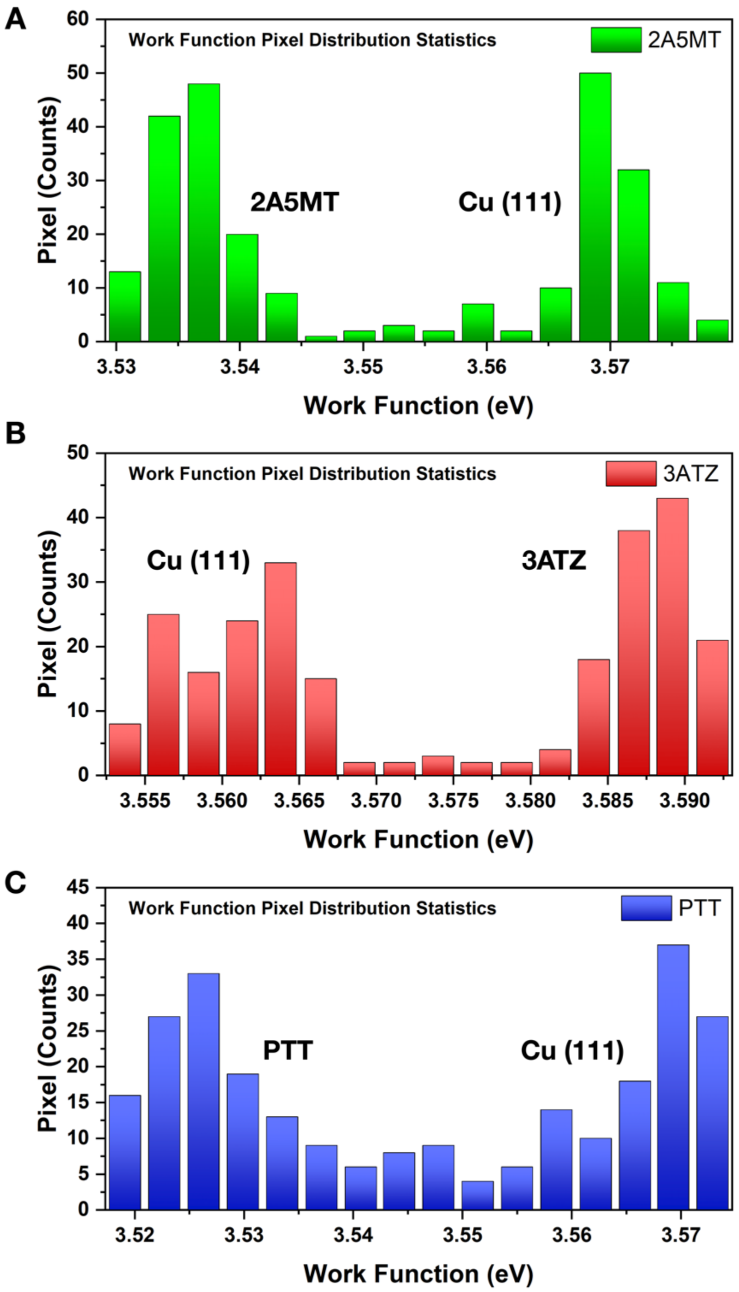

Figure S13. Statistical work function pixel distribution of (a) 2A5MT, (b) 3ATZ, and (c) PTT, which indicates the heterogeneous electron tunneling behavior between Cu-S bonding and Cu-N bonding.

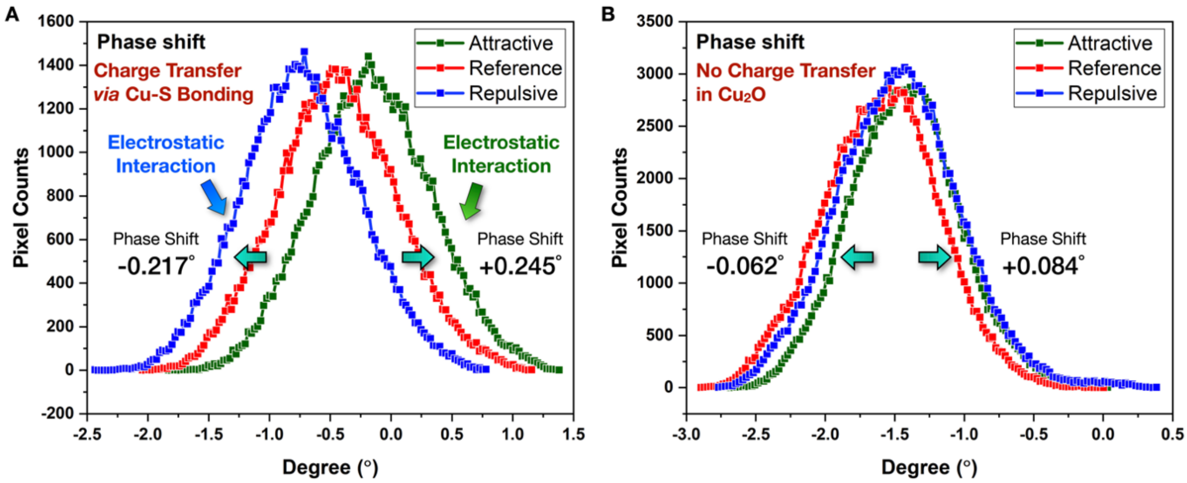

Figure S14. Phase shift at a) PTT, which anchored with Cu-S and Cu-N bonding, and b) Cu_2O, which clearly indicates the heterogeneous tip-sample electrostatic interaction.

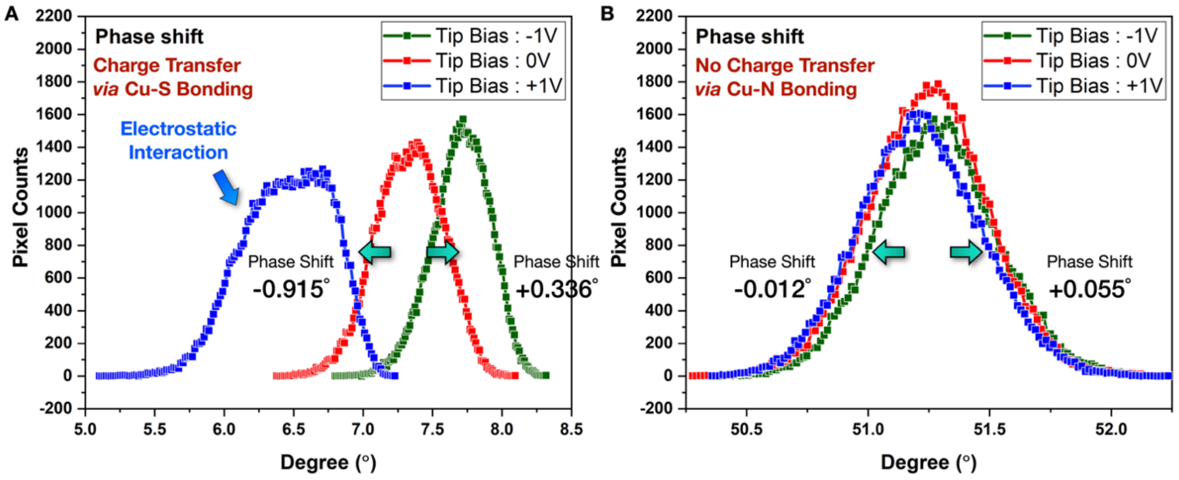

Figure S15. Tip bias effects of phase shift at a) sulfur-anchored domain and b) nitrogen-anchored domain, which clearly indicates the heterogeneous tip-sample electrostatic interaction.

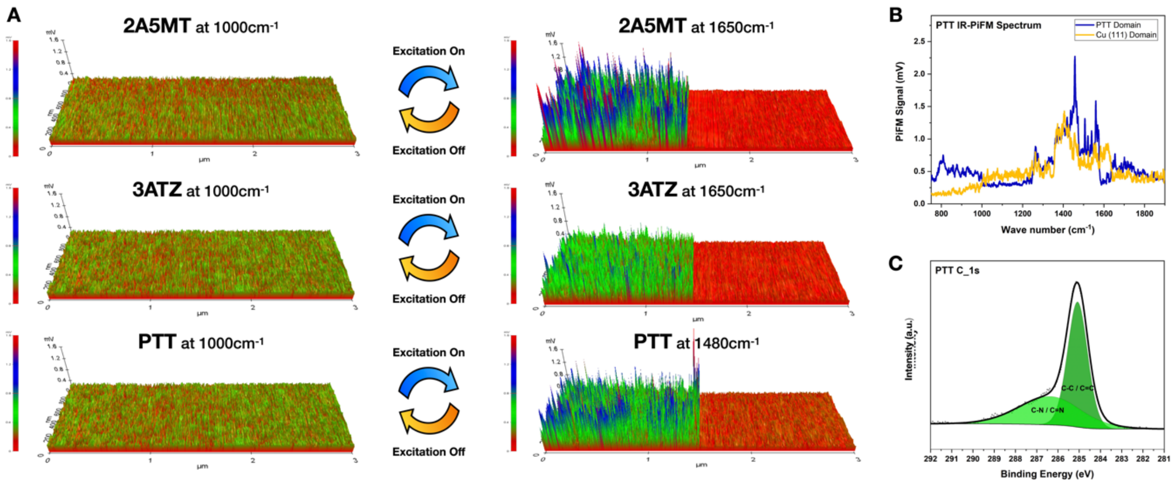

Figure S16. a) PiFM spatio-spectral imaging with IR-excitation switching, which was spatially correlated with heterogeneous IR spectra intensity. Line profile b) IR spectra of PTT and Cu (111) wafer and c) XPS C 1s peak, exhibiting the nanoscale chemical characteristics of PTT.

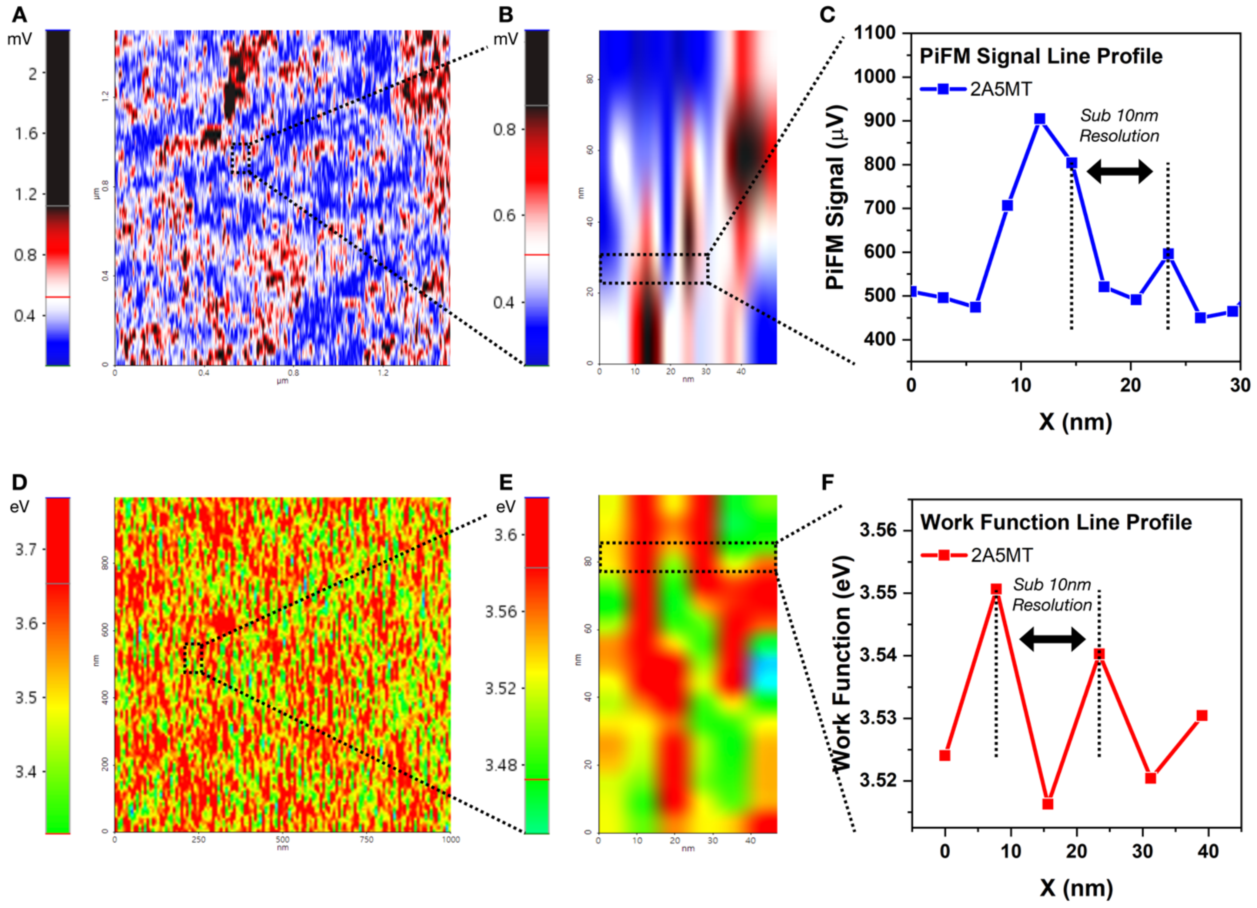

Figure S17. Spatial resolution calculation of KPFM and PiFM imaging. a) 2A5MT PiFM image, b) Local 2A5MT PiFM image and c) line profile, which indicates the PiF heterogeneity, and d) 2A5MT KPFM image, e) Local 2A5MT work function image and f) line profile, which indicates the work function heterogeneity.

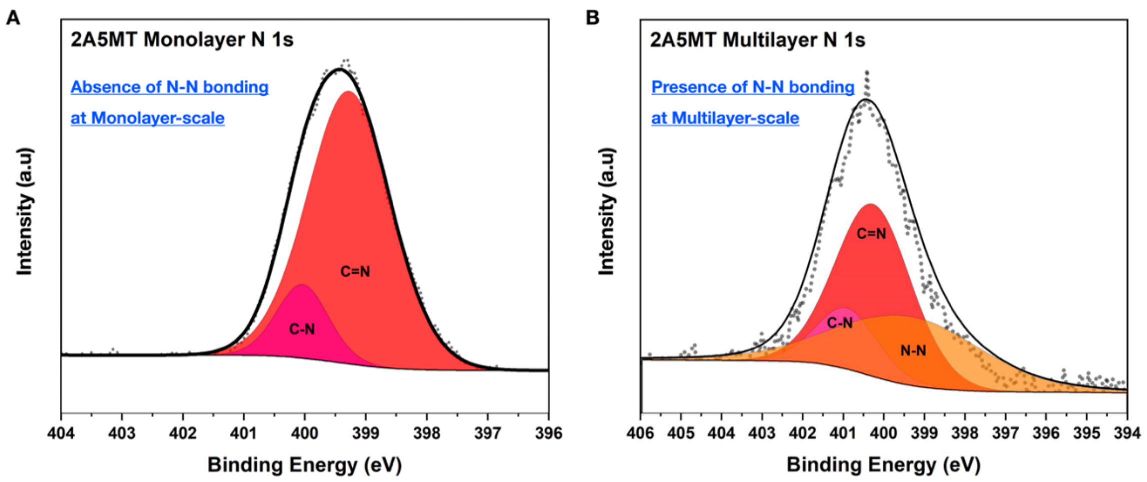

Figure S18. XPS N 1s spectra of a) 2A5MT monolayers and b) 2A5MT multilayers, which imply that the N-N bonding formation is presence in the multilayer-scale, whereas absence in the monolayer-scale.
